# Integrating Central Venous and Arterial Line Placement Training for Respiratory Therapists: A Sustainable Strategic Approach to Enhance Patient Care

**DOI:** 10.1155/ccrp/3224037

**Published:** 2025-07-28

**Authors:** Rebecca McClay, Orlando Garner, Ashley Pyle, Gerardo Catalasan, Michael Mileski

**Affiliations:** ^1^School of Science, Technology, Engineering, and Math, American Public University System, 111 W. Congress St., Charles Town, West Virginia 25414, USA; ^2^Critical Care, Midland Memorial Hospital, 400 Rosalind Redfern Grover Parkway, Midland, Texas 79701, USA; ^3^Vascular Access, Midland Memorial Hospital, 400 Rosalind Redfern Grover Parkway, Midland, Texas 79701, USA; ^4^School of Health Administration, Texas State University, 601 University Drive, Encino Hall—250, San Marcos, Texas 78666, USA

**Keywords:** advanced practice provider, arterial access, central line, respiratory therapist, vascular access

## Abstract

**Background:** This manuscript examines the use of respiratory therapists (RTs) to perform central venous and arterial line placements to address the lack of available staff to perform these procedures. To address these concerns, researchers implemented a program to provide further education to RTs to advance their skills to perform these procedures. Our facility sought to create a train-the-trainer formatted vascular access program utilizing RTs to relieve procedure burdens for critical care providers and maintain safe patient care with CLABSI rates better than the National Database of Nursing Quality Indicators (NDNQI) 95th percentile.

**Methods:** A quality improvement project using the IOWA model was performed at the mixed ICU/CCU at a West Texas tertiary care hospital. All patients admitted from May 2017 through December 2023 to the mixed ICU/CCU for arterial catheters (ACs) and all inpatient units for central venous catheters (CVCs) were included. A training program using formal evidence-based protocols was created by the critical care medical director, who implemented the program and provided the original training with the goal of educating facility RTs on proper insertion of venous and ACs. Simple descriptive statistics were used to analyze the results of the program.

**Results:** Over the 5-year retrospective review of RTs placing vascular access lines, only two negative events occurred. Our RTs performed 3878 ACs with zero complications. They also performed 6471 CVCs with only two complications (both pneumothoraces). Overall, the RT team had a success rate of 94.45% There was a minimal complication rate of 0.03%.

**Conclusions:** We found the integration of RTs to the vascular access role to be highly successful in meeting both facility and patient needs.

**Summary**

• RTs are very effective in placing CVC and AC in the ICU/CCU.
• RTs should be a strong consideration to meet facility needs where other professionals are unavailable to meet demand.
• RTs can be an effective mechanism in providing higher levels of care to ICU/CCU patients.

## 1. Introduction

### 1.1. Background

Arterial catheters (ACs) are used to monitor dynamic blood pressure and advanced cardiac indices and provide access for frequent arterial blood draws. Central venous catheters (CVCs), commonly used for various medical interventions such as desiccant fluid administration, medication delivery, and hemodynamic monitoring, are typically placed by physicians or specially trained nurses. Integrating respiratory therapists (RTs) into this process can lead to a more streamlined and collaborative approach to patient care. Rural settings often do not have qualified staff available to place catheters 24/7 [1], and increasing the pool of capable providers to provide the service can lead to an increased quality of care and a decrease in time to catheter placement [2, 3]. In response to the evolving healthcare landscape and the increasing demand for comprehensive patient care, there has been a growing recognition of the opportunity to expand the skill set of RTs [4, 5]. Traditionally, RTs have played a crucial role in managing respiratory conditions, administering inhaled respiratory therapies, performing arterial punctures, and providing care for patients requiring mechanical ventilation. Considering the translatable skill sets RTs have, they can be uptrained in placing ACs and CVCs [6, 7]. The advantage of trained RTs adhering to their scope of practice and following established protocols under providers' orders is a safe alternative to provider-placed CVCs and ACs at large acute care facilities and organizations [2, 6, 8]. Additional positive outcomes have included faster time to placement, increased provider availability for other care tasks, and lower facility costs [2, 8, 9]. RTs are available during off-hours when other qualified staff may not be readily available at the facility, making the use of them advantageous.

The rationale behind extending the credentialing of RTs to include AC and CVC placement stems from the desire to alleviate the provider burden and enhance the efficiency of healthcare delivery [9]. As the research facility was a rural serving institution, qualified staff to insert CVCs and ACs were not always readily available. A 24/7 team available to provide this service is understood to decrease the number of CLABSIs, as well as decrease expenses and improve the quality of care [2]. Several factors underscore the feasibility and potential benefits of training RTs in arterial line (AL) and central venous line (CVL) placement.

Expertise in anatomy and physiology:
  • RTs possess a comprehensive understanding of human anatomy, especially the cardiovascular and respiratory systems, and routinely use this knowledge to obtain arterial blood samples, providing a solid foundation for learning the intricacies of vascular access of AL and CVL placement [6].
Critical thinking and decision-making skills:
  • RTs are trained to think critically and make rapid decisions in high-pressure situations, making them well-suited for the dynamic nature of AL and CVL placement procedures [2, 8]. Their ability to assess and respond to changes in patient's status aligns with the complexities of this invasive intervention.
Interdisciplinary collaboration:
  • Integrating RTs into AL and CVL placement fosters interdisciplinary collaboration within the healthcare team. By working alongside physicians and nurses, RTs can contribute their unique skills to enhance the overall efficiency and effectiveness of patient care [6].
Reducing workload of physicians and nurses:
  • Given the increasing demands on healthcare providers, delegating certain procedures to RTs, such as AL and CVL placement, can help distribute the workload more evenly. This allows physicians and nurses to focus on their respective areas of expertise, ultimately improving patient outcomes [9].

### 1.2. CLABSI's and Prevention

A CLABSI is a laboratory-confirmed bloodstream infection not related to another site. This infection occurs within 48 h of line placement. It is estimated that 250K bloodstream infections occur annually [10]. CLABSIs are the costliest of all healthcare-associated infections, it is estimated to be approximately $33K per case with an associated 10% mortality rate. Gronbeck and Miller [3] examined RT placement of ACs in their facility (Kaiser Permanente Medical Center, San Rafael, CA). They found a decrease in infection rates after initiating an AC protocol despite an overall increase in the number of catheters placed. Johnson et al. [2] assessed CLABSI rates at Banner Boswell Medical Center in Phoenix, AZ, a 501-bed acute care hospital, and found that rates of CLABSI were decreased when a dedicated vascular access team was in place 24 h a day, 7 days a week, which ultimately lead to decreased expenses, increased efficiency, quality of care, and improved patient satisfaction. Ultimately, similar results were seen at our facility.

Considering the significant skill sets of RTs, the high workload burden on other healthcare providers, and the known positive outcomes from the literature, we created a program that sought to uptrain RTs in performing vascular access procedures to reduce time to CVC and AC in an inpatient setting. Simplistically, the research shows that the more staff qualified and available to provide CVC and AC insertion, the better the patient outcomes [11–13]. For the purposes of our research here, we chose to utilize RTs to insert vascular catheters as we understood their presence in the rural hospital to be 24/7. This cannot be said of all other providers. Furthermore, we chose to pursue a scenario where we focused on a larger number of qualified practitioners being available to perform the procedures, based on an already limited number of staff in our rural setting.

## 2. Materials and Methods

A quality improvement project using the IOWA model was performed at the mixed ICU/CCU at Midland Memorial Hospital, Midland, Texas, USA, a West Texas tertiary care hospital (consisting of a critical care unit and progressive care unit). All patients admitted from May 2017 through December 2023 to the mixed ICU/CCU for ACs and all inpatient units for CVCs were included. A training program using formal evidence–based protocols was created by the critical care medical director, who implemented the program and provided the original training with the goal of educating facility RTs on proper insertion of venous catheter and AC. Simple descriptive statistics were used to analyze the results of the program.

The study was largely conducted in our critical care unit (26 beds) and our progressive care unit (22 beds). The CCU is generally staffed with one intensivist physician who oversees all patient care in the unit during the day. At night, physicians are on call, and the facility has an advanced practice provider (APP and NP) on staff to provide care. This APP is often called away from the CCU to the PCU or ED to provide care or consultation, often leaving a need for other providers to administer emergent care, such as RTs completing AC and venous catheter insertions. The PCU is staffed similarly, with medical oversight being provided by the hospitalist team during the day and a nocturnist at night.

### 2.1. Outcome

The outcome of this study was the successful integration of RTs into the expanded role of practice for venous catheter insertion.

### 2.2. Data Collection

Data for this quality improvement project were collected from de-identified medical records. Researchers were able to assess whether or not patients had a venous catheter placement and whether or not there were any complications that arose from the insertion.

### 2.3. Statistical Analyses

Simple descriptive statistics were utilized to analyze the data collected.

### 2.4. Training Program

Expanding the role of RTs to include the placement of CVCs following specific protocols represents a strategic approach to address the evolving needs of the healthcare system. By capitalizing on the existing skills and expertise of RTs, this initiative aims to optimize patient care, improve efficiency, and foster collaborative healthcare practices. Training programs and guidelines should be developed to ensure RTs acquire the necessary competencies to perform AL and CVL placement safely and effectively, thus contributing to the overall enhancement of healthcare delivery. The goal of this program was to develop a vascular access program that was self-perpetuating via a train-the-trainer method. Specific methodologies are discussed in the following paragraphs surrounding the utilized parameters.

#### 2.4.1. Criteria for Vascular Access Specialist (VAS)

RTs with a minimum of 5 years' experience with 3 years of critical care (or at the discretion of the director) and arterial blood gas competencies were provided with VAS training to perform the insertion of ACs or CVCs. Twenty five central line placements established initial competence under the supervision of the critical care medical director or their designee. After completing 25 successful procedures, trainees were considered VAS. VASs were then able to supervise other RTs after 50 independent line placements. Annual competency was maintained by performing one supervised insertion by the critical care medical director or their designee annually (simulated or actual) and a minimum of 5 central line insertions at any site annually. The initial program goal was an 80% success rate. VASs not meeting this goal at any point were subject to remediation in the form of further training or removal from the program at the discretion of the cardiopulmonary medical director and multidisciplinary central line committee.

#### 2.4.2. Arterial and Central Line VAS Training Program

Training for ultrasound-guided radial and femoral AC and femoral and internal jugular CVC insertion encompassed a 5-day didactic and simulation course. The training begins with initial concept and skill introduction, with a review of the New England Journal of Medicine insertion video [14]. This video education is then followed by four different skill stations supplied with ultrasound and vascular access training mannequins. Skill station contents are defined in Table 1. Each skill station consisted of one or more required elements. The fourth skill station during training focuses on “putting it all together” with case problems, complication management, debriefing monthly for complications, postprocedure documentation, line maintenance, line discontinuation policy, benchmarking key measures (number of attempts, success rate, deep venous thrombosis rate, and CLABSI), and complications described above [15].

Line maintenance and insertion sites are monitored daily by RT in collaboration with registered nurses (RNs) for signs of infections, hematoma, bleeding, and presence of blood return. AC and CVC are left in place until no longer indicated by protocol or provider order. Patients with an identified source of infection who responded to antibiotic therapy had catheters left in place until no longer clinically indicated.

## 3. Results

From May 2017 to December 2023, our vascular access team placed 3878 ACs and 6471 CVCs. Out of the 3878 ACs placed, no complications related to the procedure were found. Out of the 6471 CVCs placed, two patients developed associated pneumothoraces, with only one of these requiring insertion of a chest tube. The RT identified both incidents immediately after line placement during their postplacement ultrasound screening protocol. No other clinically significant bleeding or complications resulted from RT line placement. As this was a retrospective records review study, we did not collect identifying information about individual patients. We only collected information regarding the need for placement of vascular catheters in patients (yes or no) and whether there were any complications (yes or no).

**Figure 1:**
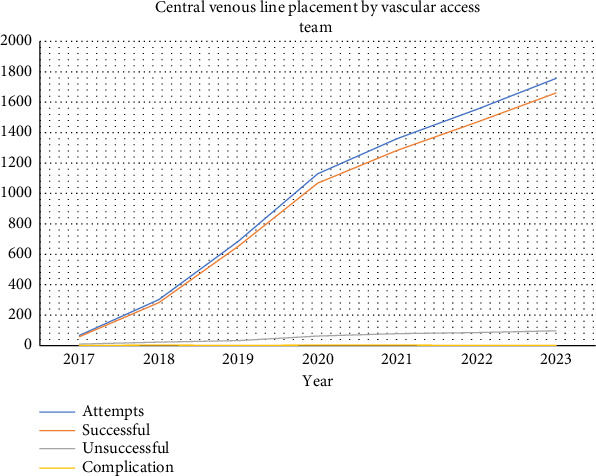
Central venous line placement by the vascular access team.

**Figure 2:**
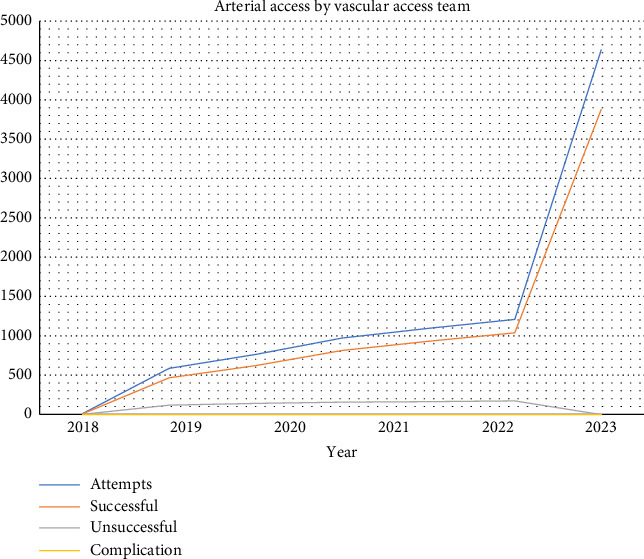
Arterial access placement by the vascular access team.

Overall, successful line placement without complications was 99.97% (Figures 1 and 2). CLABSI occurrence rate for our vascular team was 0 per 1000-line days versus 0.53 per 1000-line days by other in-house staff, and far below the target National Database of Nursing Quality Indicators (NDNQI) 95th percentile of 0.84 [16]. CLABSI occurrence rates from before the time of this study were not available for comparison. The success rate for our team was 94.45%.

## 4. Discussion

We performed a quality improvement project to reduce the time to insertion of CVCs and ACs admitted from May 2017 to December 2023. During that period, there were 3878 ACs placed without any complication related to the procedure and 6471 CVCs placed with only two instances of pneumothoraces found.

The study data show that specially trained RTs can insert ALs and CVLs safely and efficiently while practicing within the scope of their license. Ultimately, the scope of practice for any individual RT is defined by the state in which they practice. However, there is a movement toward the utilization of advanced practice RTs, being championed by the American Association for Respiratory Care (AARC), the National Board for Respiratory Care (NBRC), and the Commission on Accreditation for Respiratory Care (CoARC) [17, 18]. This is mainly to meet the needs of the large gap of available cardiopulmonary providers, including RTs [19]. This need was further supported by physicians in the field toward the utilization of advanced practice providers, including RTs, to fill the gap in meeting patient needs [20]. Ultimately, the scope of practice for an advanced practice RT is a “skilled person, qualified by academic and clinical education to provide diagnosis and treatment of respiratory diseases and disorders to patients under the supervision and responsibility of a licensed doctor of medicine or osteopathy” [21]. Furthermore, the scope of practice needs to be determined by physicians and the RT at the practice level, allowing for a flexible and customized team approach based on the practice setting [21].

The use of trained RTs for line placements provides several advantages. Unlike major hospital centers often with anesthesia and surgical residents, or other in-house physicians 24 h a day, 7 days a week, smaller community hospitals often do not have this availability of staff. An opportunity was identified to meet patient needs through novel new programs, such as the utilization of RTs to place vascular access catheters. We found that our RTs are readily available in the hospital during the night shift and on weekends when trained physicians may not be available^.^ With our training program, annual recertification, and protocol in place, RTs have been shown to have a greater success rate of initial catheter placement than physicians of various specialties and levels of training when inserting CVCs.

### 4.1. Conclusions

We developed a train-the-trainer approach to create an RT-led vascular access team to decrease work burden from other healthcare providers and improve time to access for the hospital in-patient population. We found that the rates of complications over 6 years were minimal, with only two pneumothoraces reported, which exemplifies how RTs can be adequately trained to perform these procedures. The model also allows for continued training of new personnel as a failsafe against staff turnover, while also providing a new role for RTs in the hospital.

Further studies are warranted to evaluate if the RT of vascular access success and cost-effectiveness is similar to other healthcare providers (e.g., physicians, advanced care providers, or RNs). A further assessment of cost-effectiveness for line placement could result in a decreased facility cost when comparing the time cost for an RT against that of an APP or MD for CPT 36556 for which the average Medicare payment in 2023 was $217.22 [22].

### 4.2. Limitations

Our study could not compare the VAS team's success rate against other healthcare providers at the facility due to the way data are captured for this population (i.e., CPT codes). Therefore, we cannot comment on whether our VAS success and complication rate are equivalent to that of other facility's vascular access proceduralists (i.e., physicians, nurses, and advanced care providers). This also limits our ability to establish if there are different rates of reimbursement and cost-effectiveness among these two proceduralist groups.

## Figures and Tables

**Table 1:** Skill station focus and contents.

During skill station training, RTs were also instructed on the official hospital protocol, ensuring adherence to RT licensing and scope of practice. The protocol included the following:
• Verifying a physician's order
• Obtaining informed consent
• Utilizing maximum barrier precautions
• Site selection
• Time-out procedure
• Patient preparation
• Central line insertion using both anatomic and real-time ultrasound guidance (if unable to place the line after three attempts, VAS should inform the attending physician for backup or supervision)
• Suturing the catheter
• Placement of antimicrobial disc and protective dressing (marked with date, time, and initials)
• Benchmark tool and central line bundle checklist must be performed
• Any physician or radiologist to confirm placement and absence of pneumothorax using chest X-ray and a clear line for use
• Procedure documentation
• Document line maintenance
• Discontinuation policy

## Data Availability

The data that support the findings of this study are not openly available due to reasons of sensitivity and are available from the corresponding author upon reasonable request.
