# Correction to “Integrating Central Venous and Arterial Line Placement Training for Respiratory Therapists: A Sustainable Strategic Approach to Enhance Patient Care”

**DOI:** 10.1155/ccrp/9872796

**Published:** 2026-06-02

R. McClay, O. Garner, A. Pyle, G. Catalasan, M. Mileski, “Integrating Central Venous and Arterial Line Placement Training for Respiratory Therapists: A Sustainable Strategic Approach to Enhance Patient Care,” *Critical Care Research and Practice*, 2025, 3224037, https://doi.org/10.1155/ccrp/3224037.

In the article, errors were identified in the reported numbers of central venous catheters (CVC) and arterial catheters (AC) procedures throughout the article. Specifically, the number of CVCs performed with only two complications is incorrectly reported as 6471 and should be 1655, and the number of ACs performed with zero complications is incorrectly reported as 3878 and should be 831. Please find corrected versions of Figures 1 and 2:

**FIGURE 1:**
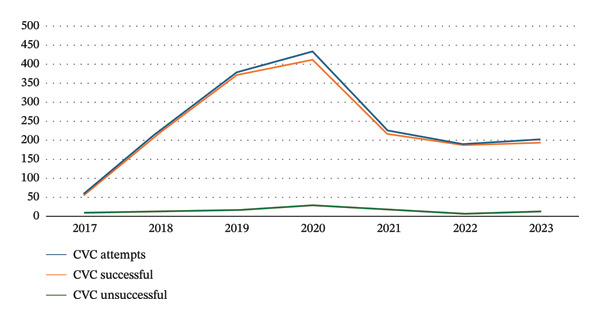
Central venous line placement by the vascular access team.

**FIGURE 2:**
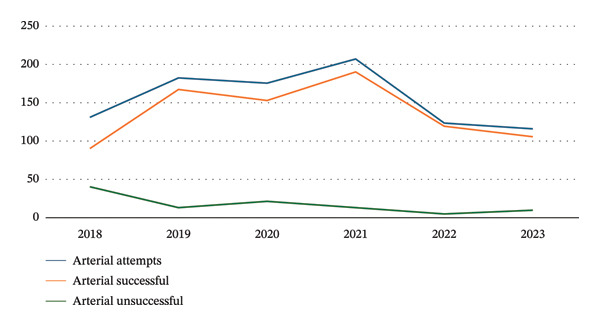
Arterial access placement by the vascular access team.

We apologize for these errors.

